# Predators modify the temperature dependence of life‐history trade‐offs

**DOI:** 10.1002/ece3.4381

**Published:** 2018-08-07

**Authors:** Thomas M. Luhring, Janna M. Vavra, Clayton E. Cressler, John P. DeLong

**Affiliations:** ^1^ School of Biological Sciences University of Nebraska ‐ Lincoln Lincoln Nebraska

**Keywords:** allocation, fecundity, fitness, phenotypic plasticity, predation, reproduction, thermal reaction norm

## Abstract

Although life histories are shaped by temperature and predation, their joint influence on the interdependence of life‐history traits is poorly understood. Shifts in one life‐history trait often necessitate shifts in another—structured in some cases by trade‐offs—leading to differing life‐history strategies among environments. The offspring size–number trade‐off connects three traits whereby a constant reproductive allocation (*R*) constrains how the number (*O*) and size (*S*) of offspring change. Increasing temperature and size‐independent predation decrease size at and time to reproduction which can lower *R* through reduced time for resource accrual or size‐constrained fecundity. We investigated how *O, S,* and *R* in a clonal population of *Daphnia magna* change across their first three clutches with temperature and size‐independent predation risk. Early in ontogeny, increased temperature moved *O* and *S* along a trade‐off curve (constant *R*) toward fewer larger offspring. Later in ontogeny, increased temperature reduced *R* in the no‐predator treatment through disproportionate decreases in *O* relative to *S*. In the predation treatment, *R* likewise decreased at warmer temperatures but to a lesser degree and more readily traded off *S* for *O* whereby the third clutch showed a constant allocation strategy of *O* versus *S* with decreasing *R*. Ontogenetic shifts in *S* and *O* rotated in a counterclockwise fashion as temperature increased and more drastically under risk of predation. These results show that predation risk can alter the temperature dependence of traits and their interactions through trade‐offs.

## 1 INTRODUCTION

Many traits (e.g., body size) show fairly predictable thermal reaction norms that may influence how organisms respond to changes in climate (Kingsolver & Huey, 2008) and that provide insight into how well trait plasticity can accommodate temperature shifts (Seebacher, White, & Franklin, 2015). However, traits may respond simultaneously to multiple environmental gradients and are often linked through trade‐offs, constraining the possible range of values that traits may take. Whether offspring size declines with increasing temperature (Atkinson, Morley, Weetman, & Hughes, 2001; Perrin, 1988), for example, may depend on the effect of temperature on the mother's size and resource acquisition, as well as the potential effect of predation risk or other interactions that also influence the optimal offspring size (Fox & Czesak, 2000; Stibor, 1992). Understanding how organism fitness responds to changing thermal regimes therefore requires investigating how life‐history traits—and the trade‐offs that constrain them—change in response to the joint effects of temperature and ecological interactions.

Predation risk has strong and often predictable effects on life histories (Benard, 2004; Culler, McPeek, & Ayres, 2014; Reznick & Endler, 1982; Van Buskirk & Schmidt, 2000; Walsh, Cooley, Biles, & Munch, 2014) and can change the temperature dependence of individual traits and fitness (Culler et al., 2014; Luhring & DeLong, 2016). However, phenotypically plastic responses to predation risk are often context specific whereby trait responses are governed by the nature of the threat (Beckerman, Rodgers, & Dennis, 2010; Benard, 2004; Bourdeau, 2009; Relyea, 2001; Riessen, 1999). For example, when predation risk is negatively size dependent and offspring mortality is relatively high compared to that of adults, freshwater snails (Physella virgate) delay reproduction to grow to a size refuge from predation (Crowl & Covich, 1990). Similarly, when exposed to cues of predators that selectively forage on larger prey (positively size‐dependent predation), cladocerans change their life‐history strategies to increase reproductive output, reproduce earlier, and at a smaller size (Beckerman et al., 2010; Stibor, 1992). Thus the effects of predation on traits (e.g., changes in time to reproduction, offspring size, number of offspring, reproductive investment) can be counter to or complement the effects of temperature depending on the nature of the risk posed by a predator. Regardless, the effects of temperature on rates (e.g., maturation, metabolism, growth), traits (e.g., body size, total reproductive investment) and links between traits (e.g., trade‐offs) can alter the underlying ability of organisms to respond to predation.

Trade‐offs result from constraints on life‐history traits such that individuals must allocate finite resources among competing priorities (Davison, Boggs, & Baudisch, 2014; de Jong & van Noordwijk, 1992; Luhring & Holdo, 2015; van Noordwijk & de Jong, 1986; Smith & Fretwell, 1974; Stearns, 1989). For example, allocating resources to growth may reduce reproduction (Black & Dodson, 1990), and allocating resources to reproduction may reduce survivorship (Kirkwood & Rose, 1991). Similarly, parsing a fixed total reproductive investment among offspring results in a central life‐history trade‐off whereby increasing the number of offspring requires a reduction in offspring size (Fox & Czesak, 2000; Lim, Senior, & Nakagawa, 2014; Rollinson & Rowe, 2015; Smith & Fretwell, 1974). This trade‐off arises because a fixed reproductive investment (*R*) in offspring biomass is given by the product of offspring size (*S*) and offspring number (*O*) (4), such that

(1) $$S\;=\;\frac{R}{O}$$

**Figure 1:**
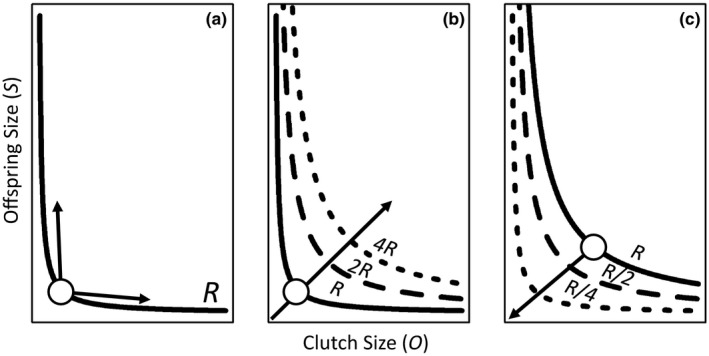
Potential outcomes for changes in allocation of resources (*R*) into number (*O*) and size (*S*) of offspring. Given a constant *R*, any change in *O* results in a change in *S* (a). If *R* increases (b) or decreases (c), the trade‐off curves move (dashed lines). Arrows in (b) and (c) indicate a constant ratio of *O*:*S* across changes in *R* (i.e., constant allocation strategy)

Although a constant reproductive investment (*R*) imposes the size–number trade‐off (Figure 1a), changing *R* would permit simultaneous increases (Figure 1b) or decreases (Figure 1c) in *O* and *S*. Thus *O* and *S* are simultaneously determined by allocation strategy (location on the trade‐off curve) as well as *R* (location of the trade‐off curve). Furthermore, many traits that are expected to be locked in trade‐offs and negatively correlated (e.g., *O, S*) are often positively correlated because of changes in the underlying *R* being partitioned (van Noordwijk & de Jong, 1986).

The size–number trade‐off may constrain options for responding to changes in the thermal environment because *O*,* S*, and *R* vary with environmental temperature (Atkinson et al., 2001; Berger, Walters, & Gotthard, 2008; Ernest et al., 2003; Perrin, 1988) while simultaneously responding to ecological interactions such as the presence and types of predators in the environment (Riessen, 1999). *R* can increase with increasing temperatures because of greater resource uptake rates or resource productivity (Burnside, Erhardt, Hammond, & Brown, 2014; Englund, Öhlund, Hein, & Diehl, 2011; Ernest et al., 2003; Kerkhoff, Enquist, Elser, & Fagan, 2005) but only up to a point (Hammond & Diamond, 1997), thus moving the trade‐off curve up and to the right at warmer temperatures (Figure 1b). However, temperature accelerates reproductive schedules which generally leads to smaller adult size at reproduction, less time to accrue *R*, and would thus require a decrease in *S* and or *O* (Kingsolver & Huey, 2008; Perrin, 1988; Walls & Ventelä, 1998; Figure 1c). How temperature will affect the offspring size–number trade‐off is thus contingent on how suites of interdependent traits jointly respond to temperature.

In this study, we assess the joint effects of temperature and predation risk on the plasticity of the offspring size–number trade‐off in *Daphnia magna*. *Daphnia* show phenotypically plastic changes in *O* and *S* with changes in temperature and predation risk (Riessen, 1999; Walls & Ventelä, 1998). Previous work on the effects of temperature on *Daphnia* suggests that adult size at first reproduction, clutch size, and offspring size should decline with increasing temperature (Giebelhausen & Lampert, 2001). Like increasing temperature, size‐independent predation decreases adult size at and time to reproduction, however, it also increases offspring number (Riessen, 1999). We exposed *D. magna* to size‐independent predation risk (kairomones from predation of conspecifics by odonate larvae) across a 22°C range of temperatures (11–33°C) to test how *R* (total clutch biomass), *O*, and *S* change with temperature and predation risk across ontogeny (first three clutches). Specifically, we test whether predation alters the temperature dependence of *D. magna* life‐history strategies by (a) moving offspring size and number along a trade‐off curve (constant *R,* changing *O* and *S*; Figure 1a), (b) moving offspring size and number across trade‐off curves by changing *R* while maintaining a constant allocation strategy (constant *O*:*S* ratio), or (c) by simultaneously changing *R* and allocation strategies (changing *O:S* ratio).

## 2 MATERIALS AND METHODS

### 2.1 Study organism and husbandry

*Daphnia magna* clones were maintained in 30‐ml glass vials (Fisherbrand Shell Vial, 8 dram) containing 25 ml of COMBO media (Kilham, Kreeger, Lynn, Goulden, & Herrera, 1998) absent of nitrates and phosphates to limit bacterial contamination. A climate‐controlled walk‐in cooler (US Cooler model FCR3476GLI) was utilized to maintain a constant 17°C temperature and a 16:8 light/dark cycle. *Daphnia* were fed every other day with the green algae *Chlamydomonas reinhardtii* (CPCC 243) at a concentration of 0.01 mg C/ml. Algae were cultured in 1‐L flasks containing 600 ml of COMBO media with nitrates and phosphates to ensure algal growth, at 23°C under a 16:8 light–dark cycle. Algal cultures were harvested on the seventh day of growth, and cell density was determined using an Epoch microplate spectrophotometer (BioTek Instruments). To standardize maternal effects, individual clones were maintained under the above conditions for three generations. Third brood neonates were collected within 24 h of birth and randomly assigned to experimental treatments.

### 2.2 Predator cue production

Odonate larvae (mostly libellulids) were collected from a freshwater pond on the Spring Creek Prairie Audubon Center in southeastern Nebraska (Luhring & DeLong, 2016; Novich, Erickson, Kalinoski, & DeLong, 2014). Odonates are *Daphnia* predators in freshwater systems that can exert strong predation effects under natural conditions (Burks, Jeppesen, & Lodge, 2001). Collected odonate larvae consumed the complete size range of *Daphnia magna* (hatchling to adult) in our study and thus presented a size‐independent source of predation risk. A pilot study demonstrated that *Daphnia magna* accelerated maturation rates (first date of reproduction) when presented with either fresh or frozen water from containers where odonates were fed *Daphnia magna*. Prior to the experiment, *Daphnia magna* of various sizes and ages (*N *=* *340) were placed in 2 L of COMBO with a community of small (~10–15 mm total length) odonates (*N *=* *17). This was repeated across six containers for each night of cue production. Predators were allowed 24 hr to consume prey and produce a variety of kairomone sources (feces, excretion, etc.). After the 24 hr, the predator cue water was filtered through 63 μm sieves and predators saved for subsequent cue production. The cue water from all containers within a night (hereafter “batch”) was combined, mixed and then immediately frozen in 50–200‐ml increments to prevent cue degradation (Crawford, Hickman, & Luhring, 2012; Hickman, Stone, & Mathis, 2004). Three total batches of predator cue were prepared in this manner. For each water change during the experiment, equal amounts of predator cue water from each batch (1 L) were slowly thawed in lukewarm water baths and then combined to produce a master mix (3 L) of predator cue so that all batches were equally represented within and across all water changes.

### 2.3 Husbandry and measurements during experiment

Water changes were conducted every Monday, Wednesday, and Friday starting with day 1 (Monday). During each water change, control treatments received 25 ml of fresh COMBO mixed with algae at 0.01 mg C/ml, while predator cue treatments received 25 ml of thawed predator cue water mixed with algae at 0.01 mg C/ml. All vials were acid‐washed and oven dried to prevent unintentional transfer of predator kairomones. We controlled food availability by running all experiments in 24 hr dark which prevented algal growth (Cressler, Bengtson, & Nelson, 2017).

### 2.4 Experimental design

During the first 7 days of the experiment (hereafter “natal” period), all *Daphnia* were maintained at 17°C (historic colony temperature) across seven environmental chambers (Percival Intellus Ultra Control System). After water changes on day seven, the “thermal performance curve” period (hereafter “TPC” period) began and temperatures in the seven environmental chambers were changed to 11, 17, 23, 27, 29, 31, or 33°C (chambers were randomly assigned temperatures). This temperature range encompasses both a decrease in temperature from the natal environment and a realistic increase in temperatures experienced by mobile plankton in freshwater systems (Kremer, Fey, Arellano, & Vasseur, 2018). The 7 day acclimation period was used because it allowed us to study the effects of temperature on life history across a wider range of temperature; individuals exposed from birth to the lowest temperature would not have reached sexual maturity within the experimental time horizon, whereas individuals exposed from birth to the highest temperatures have very low survivorship. Moreover, the acclimation period allowed us to isolate the effects of predation risk on age and size at maturity, key life‐history traits known to respond to predation and to subsequently influence offspring size and number (Riessen, 1999). Within each environmental chamber, four treatments were replicated 10 times, with each replicate being a single individual *Daphnia magna* housed in a 30‐mL glass vial. Treatments varied in the timing of predator cues: (a) Control—no predation cue applied during either period, (b) Early—predation cue present during the natal but not TPC period, (c) Late—predation cue absent during natal period and present during TPC period, and (d) Constant—predation cue present during natal and TPC periods.

### 2.5 Data collection

Daily observations for broods occurred until day eight (just prior to the first offspring being born), after which individuals were checked twice daily on days without water changes (30 checks over 21 days). Clutch size counts were collected during water changes to minimize handling of the adults. Offspring appearing in vials in successive checks were combined into one clutch estimate and given the earliest observation date as time of birth. Three offspring from each clutch were photographed with a Canon Vixia HFM52 camcorder attached to a Zeiss Stemi 2000‐CS dissecting microscope. Images were measured with ImageJ software (https://imagej.nih.gov/ij/) using a known pixel to μm conversion for each fixed magnification setting on the scope (calibrated daily). Length was recorded by drawing a straight line from the top of the head, directly above the eyespot, to the base of the tail spine. Because not all offspring were measured on their date of birth, we corrected for growth between date of birth and date of measurement for each clutch date by regressing back to offspring sizes from clutches within the same treatment combination that was measured on their actual date of birth. The corrected lengths were then used for calculating offspring size in μg (dry mass) by using a length to weight conversion for *D. magna* (Ebert, 1993):

$$\text{DM}\;\left(\mu g\right)\;=\;7.935\;\times \;\text{length}\;{\left(\text{mm}\right)}^{2.568}$$

Three offspring from each clutch were measured and the average size was used for a clutch estimate. Total clutch biomass was calculated as the product of clutch size and average offspring biomass for that clutch. Adults were measured in the same manner and on the same days as offspring with a Leica IC80 HD camera attached to a Leica M165C dissecting microscope. Because many births occurred on days when adults were not measured, we estimated adult size on these days by interpolating between adult sizes on days immediately before and after the clutch date.

### 2.6 Curve fitting

To understand the effects of temperature and predation risk on individual traits in the size–number trade‐off, we analyzed offspring size, clutch size, adult size at reproduction, and time to reproduction across temperature within each treatment. Adult size at each clutch was incorporated in statistical models to control for body size variation in resource accumulation (Cressler et al., 2017; van Noordwijk & de Jong, 1986) and packing constraints (Glazier, 2000). Because temperature‐dependent biological phenomena are often nonlinear (Amarasekare & Savage, 2012; DeLong et al., 2017; Kingsolver, 2009) we fit all temperature‐dependent processes with generalized additive models (GAMs) with the ‘gam’ function (‘mgcv’ package; R Core Team, 2017; Wood, 2006, 2015). Preliminary models indicated that three knots were optimal for all response variables and that temperature had strong nonlinear effects (significant smoothers). Offspring size (*S*) and clutch size (*O*) were both analyzed as dependent variables in GAMs with a nonlinear temperature effect (three knots), treatment effect, a temperature‐dependent treatment effect, time to reproduction, a temperature‐dependent time to reproduction effect, adult size, and the temperature‐dependent effect of adult size. Time to reproduction and adult size at reproduction were analyzed in GAMs with nonlinear temperature effects, treatment effects, and temperature‐dependent treatment effects. GAMs were checked with the ‘gam.check’ function prior to analysis of main effects and smoothing terms (temperature‐dependent effects of treatment, time to reproduction, and adult size at reproduction) through ‘anova.gam’ in the ‘mgcv’ package.

We only analyzed first clutches that were produced prior to day 11 (4 days after TPC performance period started; *N *=* *124 replicates) because we were interested in the signature of conditions of the natal environment (variation in predation regimes at the colony temperature of 17°C) on life‐history traits. We chose day 11 as it was the earliest day for which first clutches were produced in all treatment‐by‐temperature combinations except 11 and 33°C. Not all treatments produced offspring at 11 and 33°C, and therefore these temperatures were removed from curve fitting analyses. Second and third clutches developed entirely within the TPC period and were not restricted by experimental day prior to analysis. Clutch size and offspring size analyses were restricted to clutches with more than one offspring, as clutches of size one were generally partial clutches from unhealthy individuals and were outliers relative to other replicates within treatment‐by‐temperature combinations.

### 2.7 Depicting changes in offspring size and number

To track the effects of temperature and predation risk on offspring size and number, we plotted the offspring size and number averages for each temperature against each other with three reference trade‐off curves. Along each curve, *R* remains constant (at either the average total clutch biomass at 17^°^C, ½ of that average, or ¼ of that average), while *S* and *O* change accordingly (see “*Effects of Temperature and Predation on Size–Number Trade‐offs”*). Points that vary primarily along a trade‐off curve (an inverse curve according to Equation (1)) indicate that temperature does not alter reproductive investment but simply shifts the constrained combinations of offspring size and number (H1), whereas those that traverse trade‐off lines demonstrate changes in reproductive investment (*R*), causing a shift in the trade‐off curve itself (H2, H3). The direction of movement across trade‐off curves illustrates the relative contribution of changes in *O* or *S*, with primarily horizontal or vertical movements illustrating independent changes in either *O* or *S*, respectively.

### 2.8 Temperature‐dependent shifts in ontogenetic patterns of O:S

To track the effects of temperature on the patterns of *O* and *S* across ontogeny, we calculated the average trajectory from the first clutch's average value of *S* and *O* within each temperature (17–31°C) to the average of the second and third *O* and *S* values. We then plotted those trajectories as arrows with the origin of each arrow in *O S* space as the first clutch and the end of the arrow as the average of the second and third clutch's *O* and *S*.

## 3 RESULTS

### 3.1 Temperature and predation effects on offspring size and number

**Figure 2:**
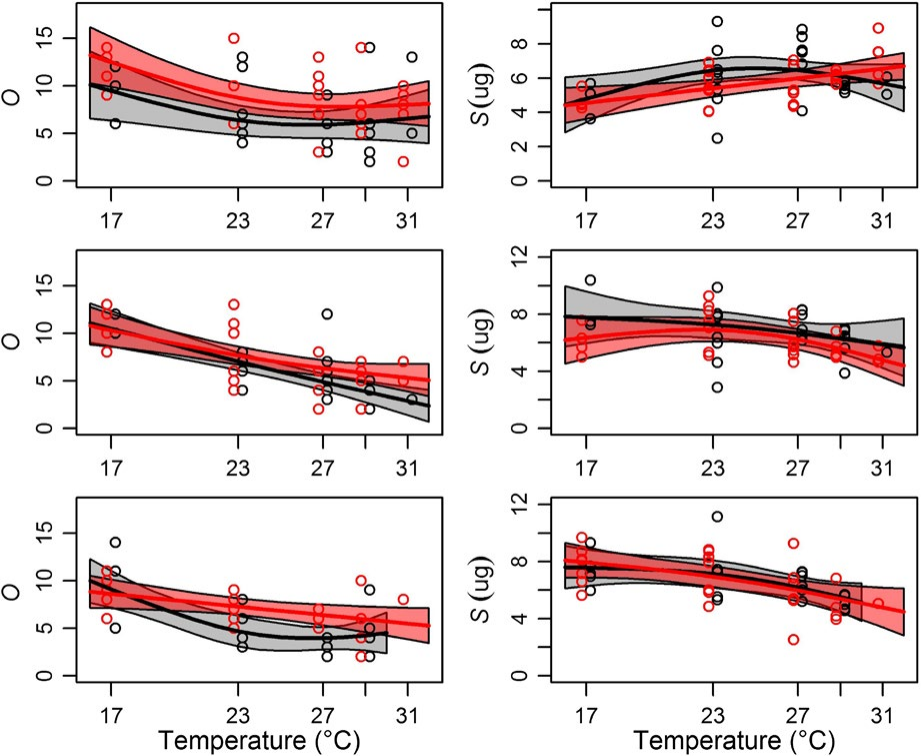
Nonlinear changes in *O* (clutch size) (left) and *S* (offspring mass) (right) across clutches (top to bottom clutches 1–3). The control treatment is indicated by black and constant predation treatment is indicated by red. Individual *Daphnia* clutches are indicated by dots and overlaid by a fitted gam (solid line) with 95% confidence bands (highlighted areas surrounding each line) [Colour figure can be viewed at http://wileyonlinelibrary.com]

Temperature (either alone or in combination with another predictor) had nonlinear effects on *S*,* O*, adult size at reproduction, and time to reproduction for all three clutches (Table 1). Clutch size (*O*) generally decreased with temperature and increased with predation risk in all three clutches (Figure 2, Table 1; Supporting information Figure S1). Offspring size (*S*) showed a positive relationship with temperature for the first clutch, but shifted to a negative size–temperature relationship (Kingsolver & Huey, 2008) once temperature exposure of the adults was more chronic (clutches 2, 3; Figure 2, Table 1). Predation effects on *S* were inconsistent and largely absent except for smaller first clutch offspring in the early exposure treatment (Supporting information Figure S2). There were also treatment‐specific temperature curves for *S* in the first clutch control and third clutch constant treatments (Figure 2, Table 1).

**Table 1:** Summary table of parametric and smooth term statistical analyses for number of offspring, offspring size, time to reproduction (clutch day), and adult size at reproduction for the first three clutches

Response	Clutch	Parametric terms	*df*	*F*	*p*	Smooth terms	*Ref. df*	*F*	*p*
Number of offspring (clutch size)	1	Temperature	**1**	**61.10**	**<0.001**	Temperature	0.8	2.90	0.136
		Predation Treatment	**3**	**5.60**	**0.001**	Temp:Control	0.8	1.93	0.217
		Time to reproduction	**1**	**42.50**	**<0.001**	Temp:Early	1.6	1.19	0.176
		Adult Size at reproduction	**1**	**5.70**	**0.018**	Temp:Late	1.3	0.25	0.766
						Temp:Constant	**0.8**	**7.52**	**0.016**
						Temp:Time to Reproduction	**2.2**	**15.09**	**<0.001**
						Temp:Adult Size at Reproduction	1.5	4.39	0.123
	2	Temperature	**1**	**4.46**	**0.037**	Temperature	**0.0**	**1634.36**	**0.001**
		Predation treatment	**3**	**4.59**	**0.005**	Temp:Control	0.7	5.20	0.054
		Time to reproduction	1	0.40	0.529	Temp:Early	0.8	1.11	0.358
		Adult size at reproduction	1	1.21	0.273	Temp:Late	0.8	3.24	0.122
						Temp:Constant	0.7	0.00	0.972
						Temp:Time to Reproduction	1.5	0.23	0.797
						Temp:Adult Size at Reproduction	1.5	2.96	0.162
	3	Temperature	**1**	**24.31**	**<0.001**	Temperature	**0.8**	**50.47**	**<0.001**
		Predation Treatment	**3**	**4.18**	**0.009**	Temp:Control	**1.8**	**23.24**	**<0.001**
		Time to Reproduction	**1**	**16.74**	**<0.001**	Temp:Early	**0.8**	**39.02**	**<0.001**
		Adult Size at Reproduction	**1**	0.57	0.453	Temp:Late	**0.8**	**28.30**	**<0.001**
						Temp:Constant	**1.8**	**14.80**	**<0.001**
						Temp:Time to Reproduction	**1.5**	**20.13**	**<0.001**
						Temp:Adult Size at Reproduction	1.5	0.16	0.761
Offspring Size (μg)	1	Temperature	**1**	**38.06**	**<0.001**	Temperature	**0.0**	**217.88**	**0.002**
		Predation treatment	**3**	**4.65**	**0.004**	Temp:Control	**1.7**	**6.27**	**0.015**
		Time to reproduction	**1**	**12.53**	**0.001**	Temp:Early	0.8	4.73	0.061
		Adult size at reproduction	**1**	**7.86**	**0.006**	Temp:Late	1.6	2.64	0.137
						Temp:Constant	0.8	1.38	0.310
						Temp:Time to Reproduction	**1.5**	**6.53**	**0.038**
						Temp:Adult Size at Reproduction	1.5	6.33	0.070
	2	Temperature	1	0.58	0.450	Temperature	0.8	2.43	0.171
		Predation treatment	3	1.03	0.383	Temp:Control	1.7	2.51	0.177
		Time to reproduction	**1**	**5.66**	**0.019**	Temp:Early	0.8	0.33	0.608
		Adult size at reproduction	1	0.90	0.346	Temp:Late	0.8	2.41	0.169
						Temp:Constant	0.8	1.48	0.279
						Temp:Time to Reproduction	1.5	3.46	0.161
						Temp:Adult Size at Reproduction	**2.5**	**3.81**	**0.027**
	3	Temperature	**1**	**14.83**	**<0.001**	Temperature	**0.3**	**35.63**	**0.001**
		Predation treatment	3	0.12	0.947	Temp:Control	1.2	1.12	0.209
		Time to reproduction	1	0.30	0.587	Temp:Early	1.4	2.30	0.066
		Adult size at reproduction	1	0.08	0.782	Temp:Late	0.8	1.06	0.371
						Temp:Constant	**0.8**	**6.55**	**0.028**
						Temp:Time to Reproduction	1.5	0.12	0.848
						Temp:Adult Size at Reproduction	1.5	0.03	0.899
Time to reproduction (days)	1	Temperature	**1**	**5046.95**	**<0.001**	Temperature	**0.8**	**1119.09**	**<0.001**
		Predation treatment	3	0.44	0.726	Temp:Control	**0.8**	**11.67**	**0.003**
						Temp:Early	**0.8**	**6.18**	**0.028**
						Temp:Late	**0.8**	**19.56**	**<0.001**
						Temp:Constant	**1.2**	**18.64**	**<0.001**
	2	Temperature	**1**	**3429.35**	**<0.001**	Temperature	**0.8**	**1236.39**	**<0.001**
		Predation treatment	**3**	**6.12**	**<0.001**	Temp:Control	**1.8**	**12.83**	**0.001**
						Temp:Early	**1.8**	**5.66**	**0.035**
						Temp:Late	**0.8**	**33.58**	**<0.001**
						Temp:Constant	**1.7**	**17.01**	**<0.001**
	3	Temperature	**1**	**1833.65**	**<0.001**	Temperature	**0.8**	**658.12**	**<0.001**
		Predation treatment	**3**	**5.22**	**0.002**	Temp:Control	**1.0**	0.01	0.915
						Temp:Early	**0.8**	**16.28**	**<0.001**
						Temp:Late	**1.6**	**4.97**	**0.008**
						Temp:Constant	**0.9**	**21.69**	**<0.001**
Adult size at reproduction (μg)	1	Temperature	**1**	**1392.25**	**<0.001**	Temperature	**0.8**	**333.18**	**<0.001**
		Predation treatment	3	0.36	0.780	Temp:Control	**0.8**	**7.97**	**0.013**
						Temp:Early	1.4	1.58	0.323
						Temp:Late	0.8	3.94	0.079
						Temp:Constant	1.3	2.85	0.057
	2	Temperature	**1**	**1792.98**	**<0.001**	Temperature	**0.8**	**640.25**	**<0.001**
		Predation treatment	3	0.37	0.776	Temp:Control	**1.7**	**4.18**	**0.013**
						Temp:Early	**1.5**	**8.93**	**0.004**
						Temp:Late	1.5	0.81	0.270
						Temp:Constant	**1.2**	**7.70**	**0.007**
	3	Temperature	**1**	**1630.04**	**<0.001**	Temperature	**0.8**	**544.55**	**<0.001**
		Predation treatment	**3**	**2.96**	**0.037**	Temp:Control	1.4	2.64	0.129
						Temp:Early	**0.8**	**27.13**	**<0.001**
						Temp:Late	1.3	2.46	0.077
						Temp:Constant	0.8	4.16	0.072

Note

Significant terms (*P < *0.05) are bolded.

Our results also indicate that the timing and duration of exposure to predation risk change the temperature dependence of life‐history traits. Exposure to predation cues during the natal period (early and constant cue treatments) elevated clutch number (*O*) in the first clutch (first row Supporting information Figure S1). By the second clutch, recent exposure appeared to be more impactful as late and constant exposure treatments (but not the early exposure treatment) had higher *O* at warmer temperatures than the control (second row Supporting information Figure S1). By the third clutch, exposure to predation cue at any phase of ontogeny appeared to affect the temperature dependence of *O* by elevating it at intermediate temperatures (third row Supporting information Figure S1).

### 3.2 Effects of temperature and predation on size–number trade‐offs

**Figure 3:**
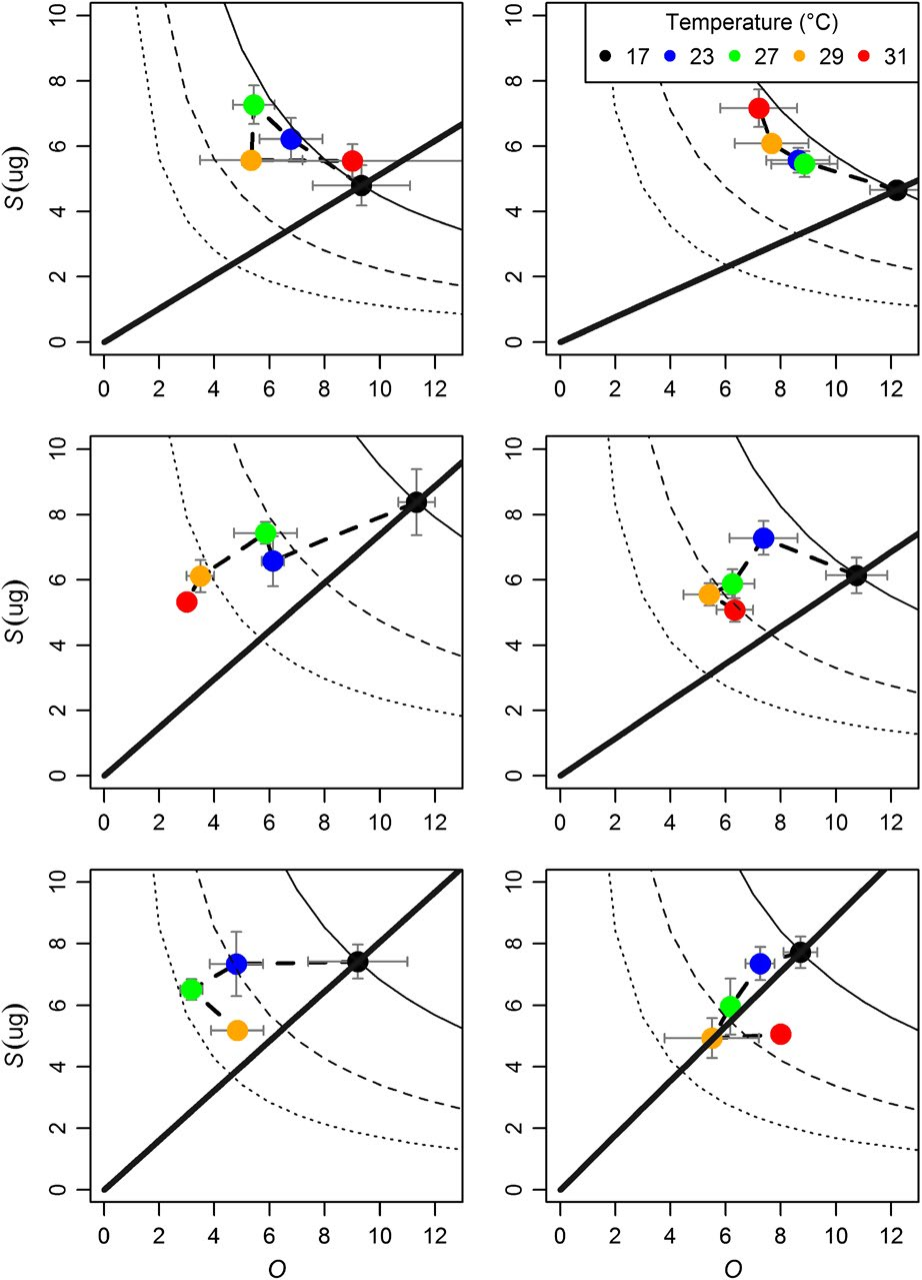
Trade‐off curves for control (left) and constant predation (right) treatments for the first three clutches of *Daphnia* (rows 1–3 correspond to clutches 1–3). The solid black curved isocline illustrates *O* versus *S* values for fixed *R* at 17°C within each treatment by clutch combination. Long dash and short dash isoclines indicate *R*/2 and *R*/4, respectively (using *R* at 17°C). Diagonal solid lines indicate a change in *R* while maintaining a constant *O*:*S* (i.e., constant allocation strategy). Each clutch within a treatment is sequentially connected by temperature (heavy dashed line) to show the temperature‐dependent change of the trade‐off across space [Colour figure can be viewed at http://wileyonlinelibrary.com]

The significant effects of temperature and predator cues on *S* and *O* led to shifts both along (constant *R*) and across size–number trade‐off curves (changing *R*; Figure 3, Table 1). Two broad patterns emerged, separated by first and later clutches. In the first clutch, *Daphnia* experienced acute temperature exposure (2.8 ± 1.7 days ±*SD*) after the 7‐day natal period, and *O* and *S* in both control and predation treatments generally followed the trade‐off curve for the first clutch (constant *R*). First clutch control *Daphnia* were spread along the trade‐off curve with no clear temperature pattern. Predation risk, however, induced a consistent response to increasing temperature where clutches moved up the offspring number–size trade‐off curve, toward fewer, larger offspring (Figure 3). Thus, reproductive investment in the first clutch was relatively constant resulting in an apparent trade‐off between *O* and *S* that moved along the trade‐off isocline (Figure 1a).

The second and third clutches were formed under more chronic exposure to temperature treatments (6.1 ± 1.7 and 10.1 ± 2.7 days after the end of the natal period, respectively). In these clutches, both *S* and *O* generally decreased across trade‐off curves (Figure 3) as *R* decreased with increasing temperatures (Figure 1c). Although increasing temperature quickly reduced *R* in control *Daphnia* in the second and third clutches (halved from 17°C to 23°C, and crossing or reaching the ¼ isocline at warmer temperatures), predation reduced the negative effects of temperature on *R* (not reaching the ½ isocline until 27°C and not reaching the ¼ isocline at any temperature). In other words, although increased temperature decreased *R* overall, *D. magna* experiencing predation risk lowered *R* more gradually than controls and maintained higher *O* relative to controls as temperature increased.

**Figure 4:**
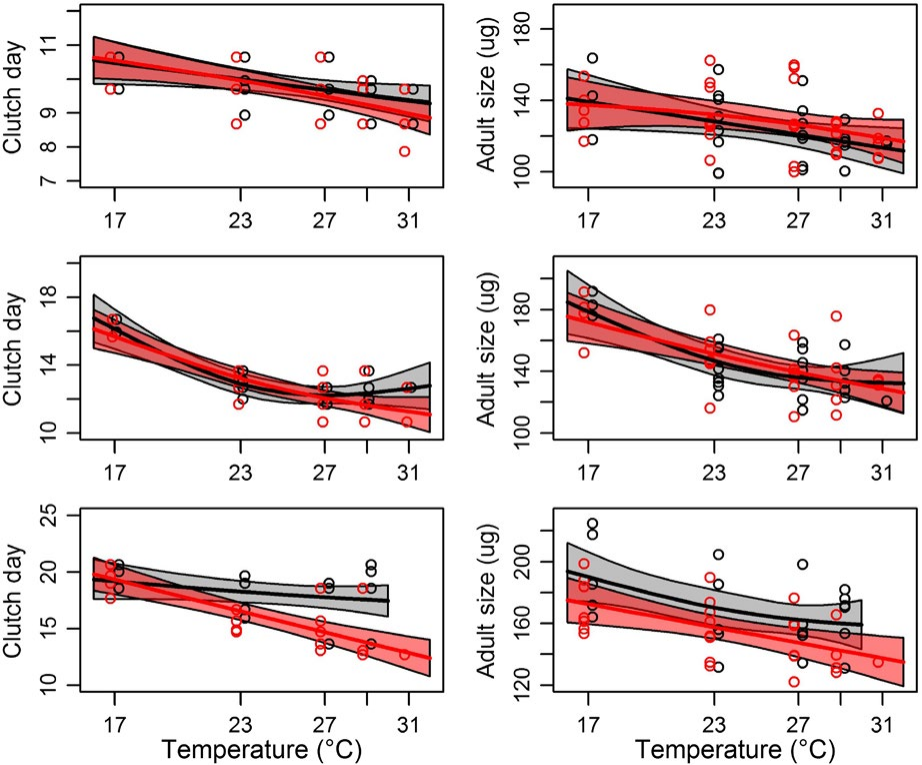
Nonlinear changes in clutch day (left) and size at reproduction (right) of adult *Daphnia* for their first three clutches (top to bottom clutches 1–3). The control treatment is indicated by black and constant predation treatment is indicated by red. Individual *Daphnia* are indicated by dots and overlaid by a fitted gam (solid line) with 95% confidence bands (highlighted areas surrounding each line) [Colour figure can be viewed at http://wileyonlinelibrary.com]

The downward movement of trade‐off curves in space with increasing temperature was consistent with accelerated reproductive schedules, and thus a reduction in *R*. The effects of temperature and predation risk on adult time and size of reproduction were prevalent across all clutches (Table 1). *Daphnia* adults reproduced earlier on average with the presence of predation cues and with increasing temperature (Table 1, Figure 4). The difference between control and constant cue treatments was most pronounced by the third clutch for both time to and size at reproduction (Figure 4), consistent with an accumulated effect of earlier clutch production over time. This increased departure from the control treatment at warmer temperatures was also seen in the early and late exposure treatments for age at reproduction (Supporting information Figure S3), indicating that reproductive schedules were influenced by both current and historical exposure to predation risk. While the temperature‐dependent nature of adult size was often nonlinear (smooth terms, Table 1), only adult size in the constant cue treatment deviated on average from *Daphnia* in the control across temperatures (Figure 4; Supporting information Figure S4).

**Figure 5:**
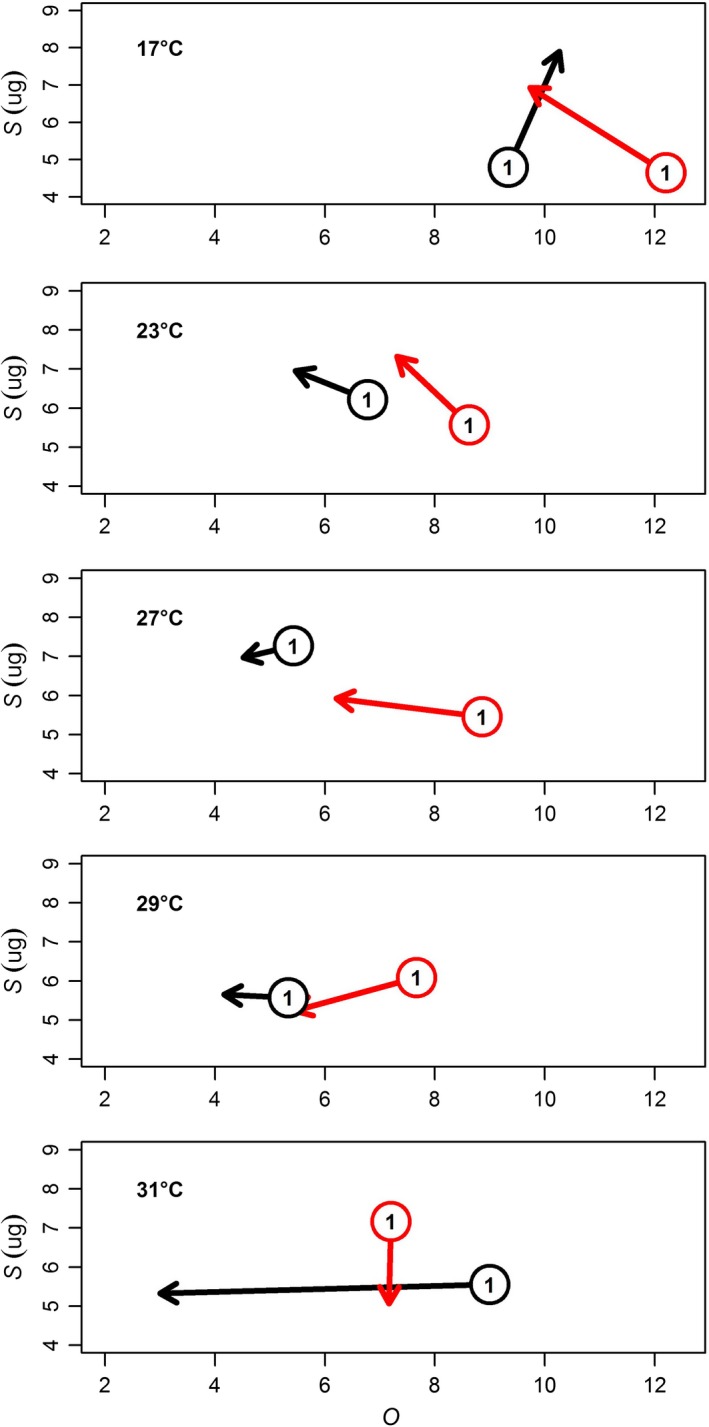
Change in ontogenetic patterns of *O* versus *S* from the first clutch (circles) to the second and third clutch (end of arrow) across temperature (17–31°C) and presence (red lines) or absence (black lines) of predation cues

Only control *D. magna* raised at their natal colony temperature (17°C) throughout the experiment increased *R*,* O,* and *S* as they progressed from their first to latter clutches (black line Figure 5). Control *D. magna* that switched from 17°C to warmer temperatures all decreased *O* to varying degrees with 31°C showing the strongest shift in *O*. *Daphnia magna* exposed to predation risk appeared to either trade‐off *O* for increased *S* (17–23°C), primarily decrease *O* (27–29°C) or *S* (31°C). Despite their difference in ontogenetic progression of *O* versus *S* at 17°C, both treatments generally progressed in a counterclockwise fashion in O versus *S* parameter space as temperature increased (Figure 5).

## 4 DISCUSSION

Organisms adjust to changing environments through shifts in life‐history strategies and traits. Thus, understanding how organisms persist in variable or shifting conditions is contingent on understanding how traits and life‐history strategies respond to the environment. Temperature‐dependent trait responses (Angilletta, Steury, & Sears, 2004; Atkinson & Sibly, 1997; Atkinson et al., 2001; Ciota, Matacchiero, Kilpatrick, & Kramer, 2014; Miin Chua, Leggat, Moya, & Baird, 2013; Orcutt & Porter, 1983; Pepin, 1991; Perrin, 1988; Sibly & Atkinson, 1994; Willott & Hassall, 1998) provide a strong link to the potential effects of climate change on ectotherms (Sinclair et al., 2016). However, life histories also respond to other contexts such as the type and strength of predation risk (Benard, 2004; Culler et al., 2014; Reznick & Endler, 1982; Van Buskirk & Schmidt, 2000; Walsh et al., 2014). And although predation risk and temperature are pervasive selective forces that shape the ecology, evolution, and phenotypic responses of organisms (Angilletta, 2009; Benard, 2004; Brown, Gillooly, Allen, Savage, & West, 2004; Kingsolver, 2009; Lima & Dill, 1990; Lind & Cresswell, 2005; Reznick & Endler, 1982; Stibor, 1992; Van Buskirk & Schmidt, 2000) their effects are generally investigated separately (but see Culler et al., 2014; Grigaltchik, Ward, & Seebacher, 2012; Luhring & DeLong, 2016). Our results indicate that shifts in life histories depend on the interaction of temperature and ecological context on multiple factors: the way in which life histories are linked (e.g., through trade‐offs), effects on constraints, and the individual's ontogenetic state.

We used the size–number trade‐off in a clonal population of *D. magna* to evaluate how a suite of linked life‐history traits responded plastically to temperature and predation risk across ontogeny (first three clutches). The size–number trade‐off helps to frame how offspring size (*S*) and number *(O*) change together given the shifting constraint of resource allocation to reproduction (*R*). All aspects of the trade‐off (*O, S, R*) simultaneously responded to predation and temperature (Figure 3, Table 1) with trait responses showing largely interactive rather than additive effects of predation and temperature (10 of 12 clutch by trait combinations showed significant treatment‐by‐temperature smoother terms in Table 1). In contrast to most previous work, we show how both the strategy (moving along the trade‐off curve; Figure 1a) and the overall allocation (moving across trade‐off curves; Figure 1b) respond to changes in temperature and predation risk across ontogeny. In this study, while *D. magna* generally had somewhat smaller offspring with increased temperature, they also showed a tendency to favor fewer large offspring as *R* decreased at warmer temperatures (located above the line showing a constant proportional decrease between *O* and *S*; Figure 3). Furthermore, as *D. magna* progressed through ontogeny they switched from constant *R* and a trade‐off mediated movement toward fewer larger offspring at warmer temperatures (potentially caused by packing constraints at smaller adult sizes; Glazier, 2000), to decreasing *R* at warmer temperatures and favoring relatively higher decreases in *O* than *S* (location above the line; Figure 3), and toward a tendency to favor a proportional decrease in *O* and *S* with increases in temperature (location along the line; Figure 3). While both control and predator‐exposed *D. magna* showed these general patterns, predation accelerated this counterclockwise movement through trait space across ontogeny (Figures 3, 5).

Life‐history strategies in our study shifted across ontogeny and the magnitude and direction of these shifts were strongly affected by temperature and predation (Figures 3, 5). Ontogenetic shifts in size–number strategy moved in a counterclockwise fashion with increasing temperature (Figure 5). However, predation risk altered 1) the location of clutches in *O S* space and 2) the direction of their movement across ontogeny at the coldest and warmest temperatures. Whereas control *D. magna* showed an ontogenetic progression toward higher *O* and *S* at 17°C, *D. magna* traded off *O* for *S* as they progressed through ontogeny. At 31°C where control *D. magna* showed a decrease in *O* for maintaining *S*, predation resulted in holding *O* constant while decreasing *S*. These results are potentially explained by shifts in reproductive strategies under perceived mortality risk from predation and temperature increases. Organisms shift reproductive strategies under changing climatic indicators of future survival (Roitberg, Sircom, Roitberg, van Alphen, & Mangel, 1993) and mortality increases exponentially with temperature (Amarasekare & Savage, 2012; Savage, Gillooly, Brown, West, & Charnov, 2004). At 17°C, *D. magna* are being held at a constant temperature throughout the experiment, whereas the remaining temperatures all represent a departure from conditions experienced during the natal period (days 1–7). Thus, control *D. magna* at 17°C reflect how *O* and *S* progress across ontogeny without added temperature or predation risk and the pattern of simultaneous increases in *O* and *S* across ontogeny (17°C controls, Figure 5) are consistent with other studies lacking these added stressors (Glazier, 1992). However, any change to temperature or predation risk completely changes the nature of how *O* and *S* change across ontogeny. This indicates that individuals embedded in food webs with heterogeneous temperatures may show very different life‐history responses than that seen in the lab under relatively benign and static conditions.

Our results highlight the temperature dependence of phenotypically plastic traits in response to predation risk and how temperature‐dependent shifts in constraints that underlie key life‐history trade‐offs shape trait space. These results serve as yet another example of the importance of incorporating multiple traits, their interactions (e.g., trade‐offs), constraints, and responses to ecologically relevant pressures (e.g., predation) into projections of how organisms will respond to climate change. Life‐history traits coevolve (Endler, 1995; Ghalambor, Walker, & Reznick, 2003; Protas et al., 2008) and respond to shifting environmental conditions through rapid evolution (Hairston, Ellner, Geber, Yoshida, & Fox, 2005; Padfield, Yvon‐Durocher, Buckling, Jennings, & Yvon‐Durocher, 2016; Thompson, 1998) and phenotypically plastic trait change (Kremer et al., 2018). The manner in which suites of life‐history traits will coevolve in response to shifting thermal clines remains is poorly understood as is the manner which these trait changes manifest across ontogeny and trade‐offs (Angilletta, 2009). Furthermore, because aquatic habitats can show strong spatiotemporal temperature variance (Kremer et al., 2018), changing location in the water column to follow food or reduce predation risk (Burks et al., 2001) may come with additional trade‐offs induced by temperature‐dependent effects on *R* and subsequently *O* and *S*. Within a period of a few days, *D. magna* shifted size and number of their offspring in response to increasing temperatures (Figure 3), and predation risk further harmonized the response direction toward fewer larger individuals. Then, across ontogeny the temperature dependence of these responses shifted as reproductive investment (*R*) showed stronger temperature dependence. Thus, within three clutches of an iteroparous organism predation risk and temperature can combine to change the nature of trait interactions from that of a trade‐off between two traits and a constant resource to that of two traits scaling with a shifting resource.

## AUTHORS’ CONTRIBUTIONS

T.M.L. conceived the study; T.M.L., J.M.V., C.E.C., and J.P.D. designed the study; T.M.L. and J.M.V. ran the experiment and collected data; T.M.L. analyzed the data. T.M.L., J.M.V., C.E.C., and J.P.D. interpreted the results; and T.M.L. drafted the manuscript. T.M.L, J.M.V., C.E.C, and J.P.D. revised and approved the manuscript. All authors contributed critically to drafts of the manuscript and gave their final approval for publication.

## CONFLICT OF INTEREST

The authors declare no competing interests.

## DATA ACCESSIBILITY

Data and R code used to generate the results and figures are available from the Dryad Digital Repository: https://doi.org/10.5061/dryad.28ms663.
