# Supplementary material for: Predators modify the temperature dependence of life‐history trade‐offs
Source: Ecol Evol. 2018 Aug 7;8(17):8818–30. doi: 10.1002/ece3.4381 (PMC6157656; doi:10.1002/ece3.4381)

Supplementary 1. Non-linear changes in clutch size in (from left to right) control, early exposure, late exposure, and constant exposure treatments across clutches (top to bottom clutches 1-3). Individual *Daphnia* clutches are indicated by dots and overlaid by a fitted gam (solid line) with 95% confidence bands (highlighted areas surrounding each line). The control treatment GAM is indicated by a dashed black line and 95% confidence bands (grey) for all treatments as a comparison. If predation cues were present during (or immediately prior to in the case of clutch 1) the GAM and 95% confidence bands are presented as red.

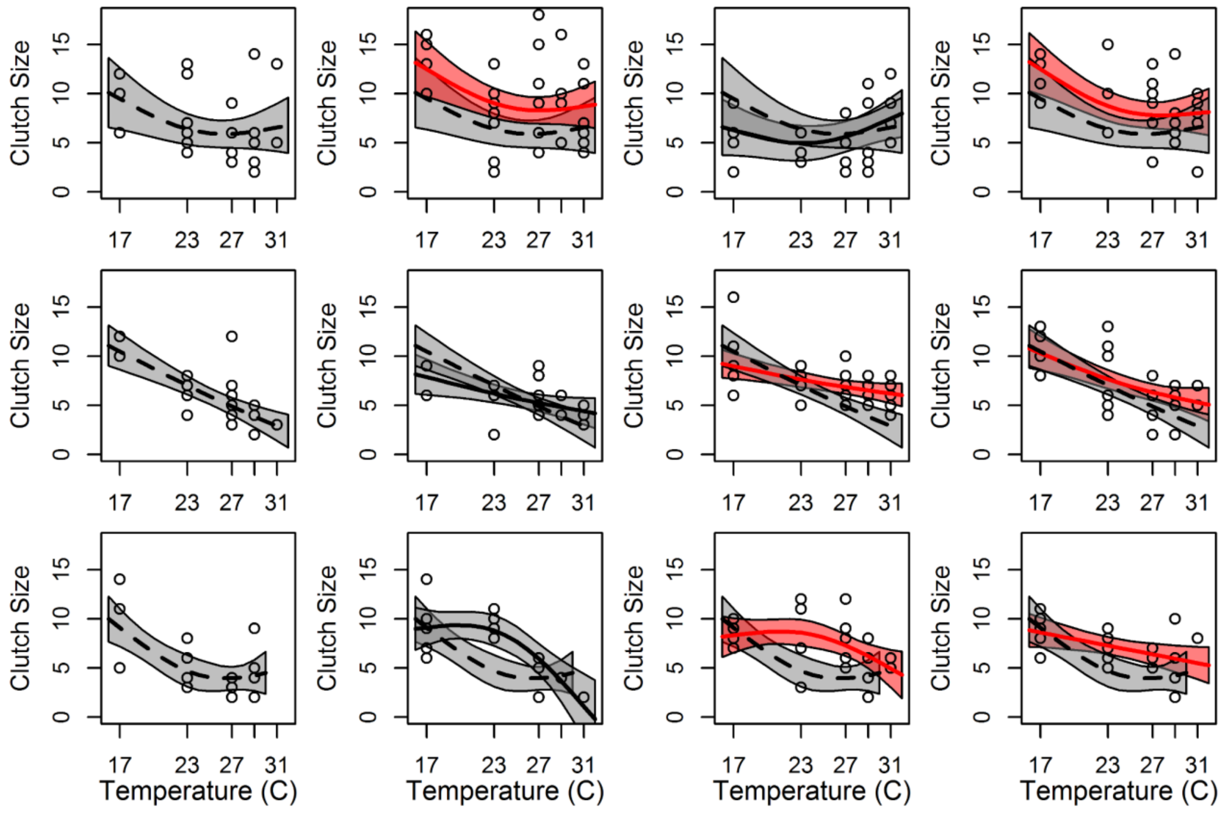

Supplementary 2. Non-linear changes in offspring size (ug) in (from left to right) control, early exposure, late exposure, and constant exposure treatments across clutches (top to bottom clutches 1-3). Mean offspring sizes for each clutch are indicated by dots and overlaid by a fitted gam (solid line) with 95% confidence bands (highlighted areas surrounding each line). The control treatment GAM is indicated by a dashed black line and 95% confidence bands (grey) for all treatments as a comparison. If predation cues were present during (or immediately prior to in the case of clutch 1) the GAM and 95% confidence bands are presented as red.

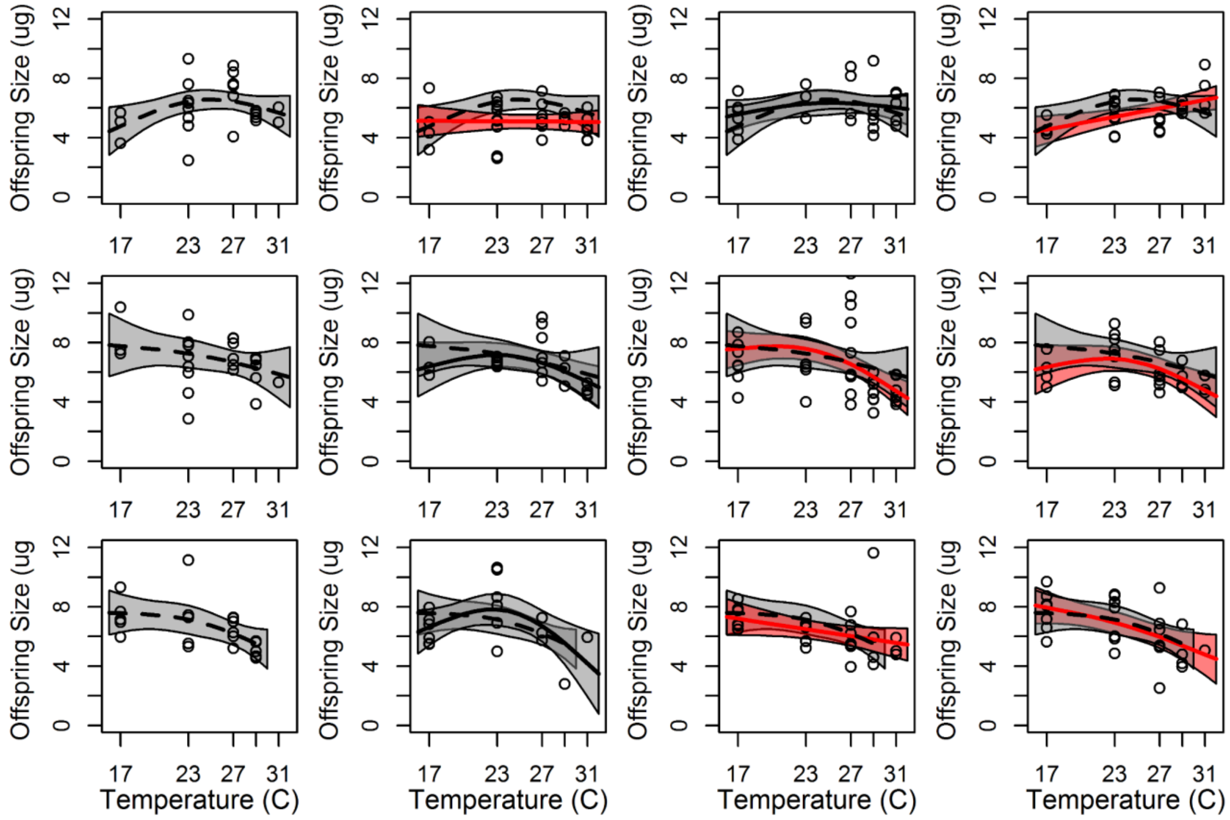

Supplementary 3. Non-linear changes in clutch date in (from left to right) control, early exposure, late exposure, and constant exposure treatments across clutches (top to bottom clutches 1-3). Individual adult ages for each clutch are indicated by dots and overlaid by a fitted gam (solid line) with 95% confidence bands (highlighted areas surrounding each line). The control treatment GAM is indicated by a dashed black line and 95% confidence bands (grey) for all treatments as a comparison. If predation cues were present during (or immediately prior to in the case of clutch 1) the GAM and 95% confidence bands are presented as red.

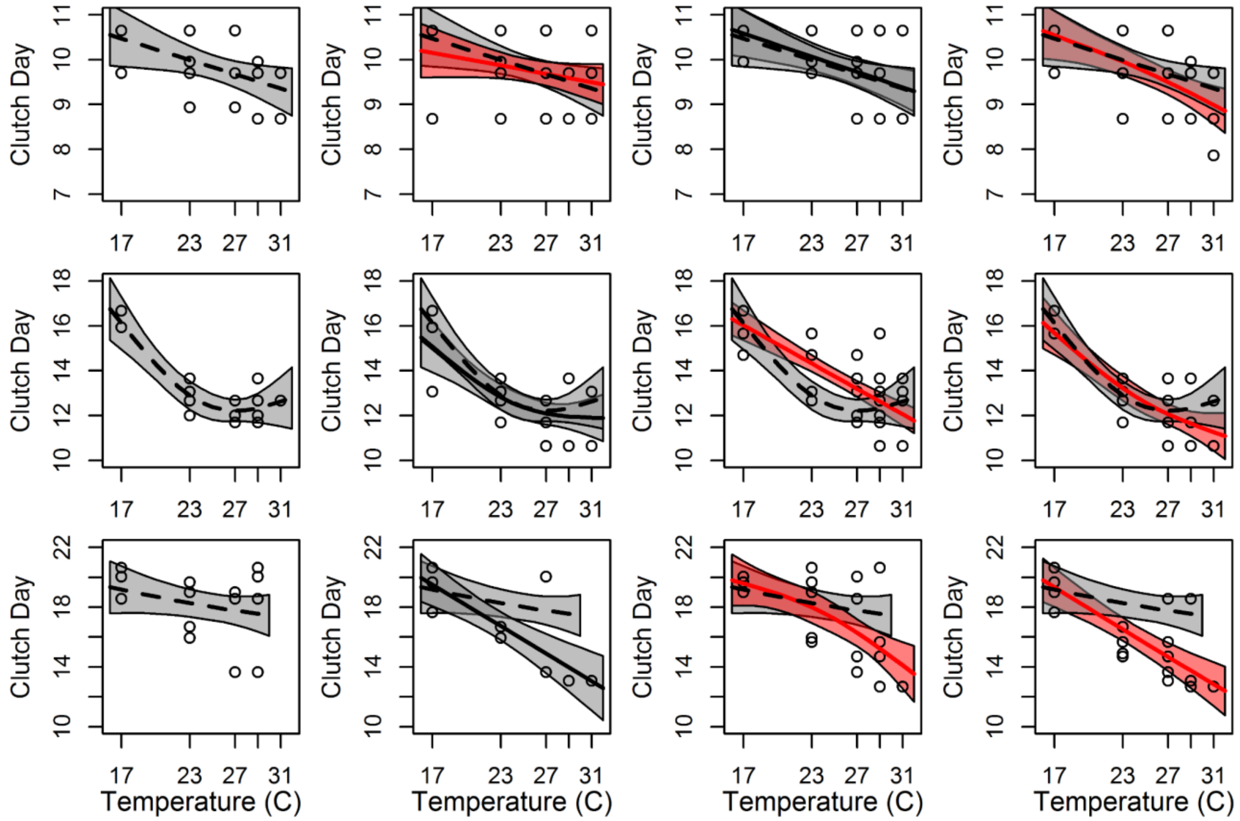

Supplementary 4. Non-linear changes in adult size at reproduction in (from left to right) control, early exposure, late exposure, and constant exposure treatments across clutches (top to bottom clutches 1-3). Individual adult sizes for each clutch are indicated by dots and overlaid by a fitted gam (solid line) with 95% confidence bands (highlighted areas surrounding each line). The control treatment GAM is indicated by a dashed black line and 95% confidence bands (grey) for all treatments as a comparison. If predation cues were present during (or immediately prior to in the case of clutch 1) the GAM and 95% confidence bands are presented as red.

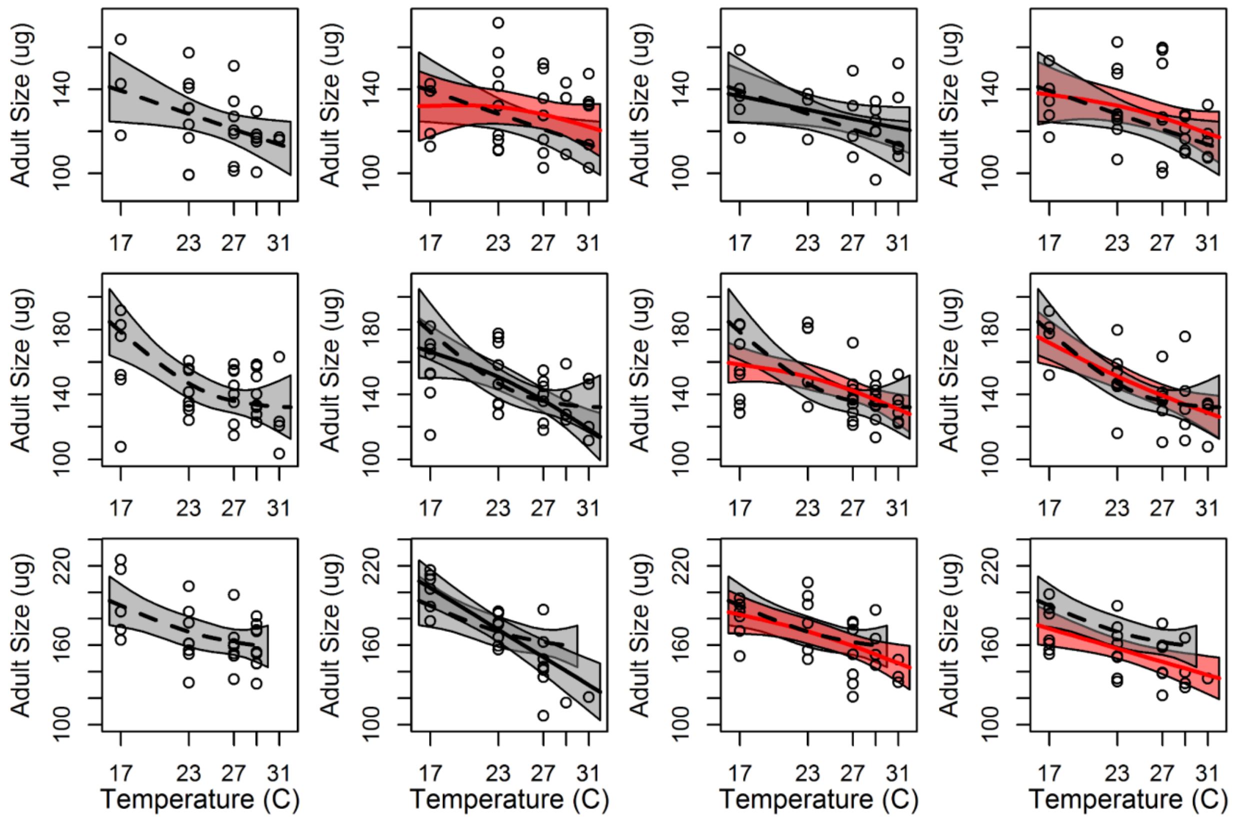

Supplement: Supplementary file 1 [file ECE3-8-8818-s001.pdf]
